# P-1849. Beta-Lactam Therapeutic Drug Monitoring in Outpatient Antimicrobial Therapy (OPAT) Patients

**DOI:** 10.1093/ofid/ofaf695.2018

**Published:** 2026-01-11

**Authors:** Kemar O Barrett, Christina G Rivera (O'Connor), Sara Ausman, Omar M Abu Saleh, Andrew D Rule, Ryan W W Stevens, Kellie N Hannan, Erin F Barreto

**Affiliations:** Mayo Clinic, Rochester, MN, Rochester, MN; Mayo Clinic, Rochester, MN; Mayo Clinic Health System - Eau Claire, Eau Claire, Wisconsin; Mayo Clinic, Rochester, MN; Mayo Clinic, Rochester, MN; Mayo Clinic, Rochester, MN; Mayo Clinic - Rochester, MN, Rochester, Minnesota; Mayo Clinic, Rochester, MN

## Abstract

**Background:**

Outpatient parenteral antimicrobial therapy (OPAT) is effective and cost-efficient, but 12%–63% of patients experience adverse events, requiring close monitoring. Therapeutic drug monitoring (TDM) is routinely used for vancomycin and aminoglycosides but is underutilized for beta-lactams (BLs) due to limited access, logistical barriers, and clinician inexperience. This study evaluated its use and clinical impact within a high-volume OPAT program.

**Figure:**
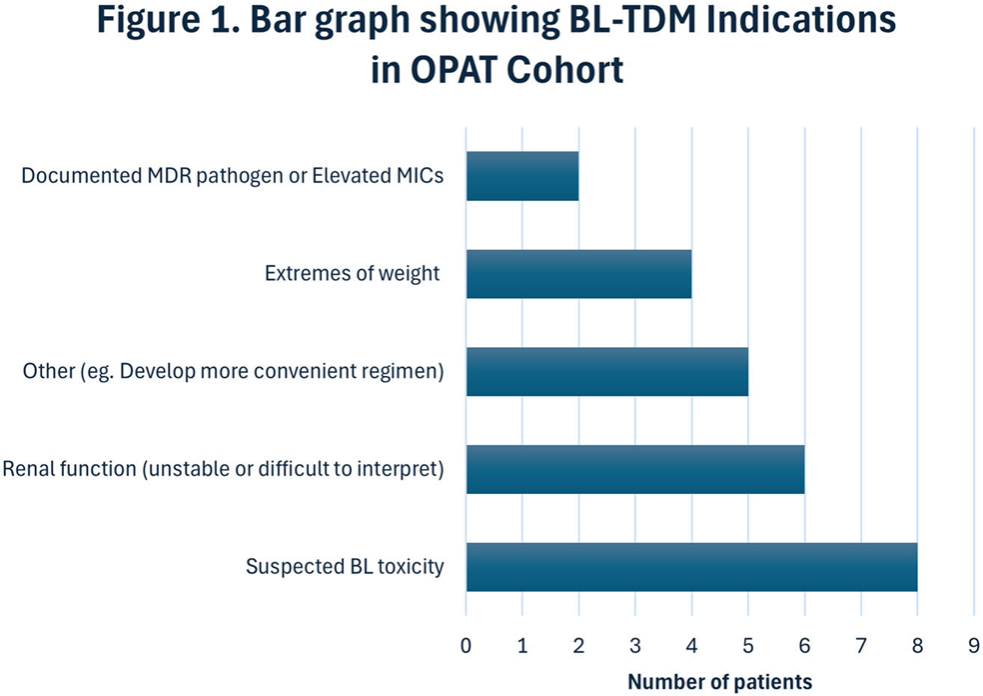

**Figure:**
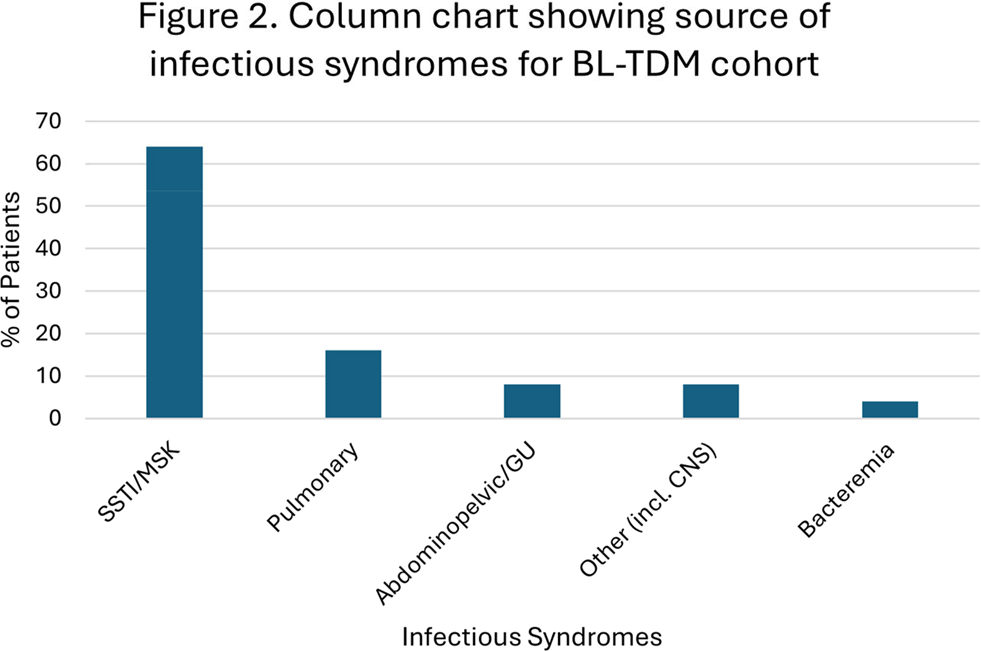

**Methods:**

This retrospective, single-center cohort included adult patients (≥18 years) enrolled in an OPAT program who received cefepime (CEF), piperacillin/tazobactam (TZP), or meropenem (MEN) and underwent BL TDM between April 2023 and April 2025. BL TDM was not protocolized within the OPAT program; however, institutional guidance supported its use in carefully selected patients. We aimed to describe TDM indications, lab trends, microbiological profiles, and the impact of TDM on treatment.

**Figure:**
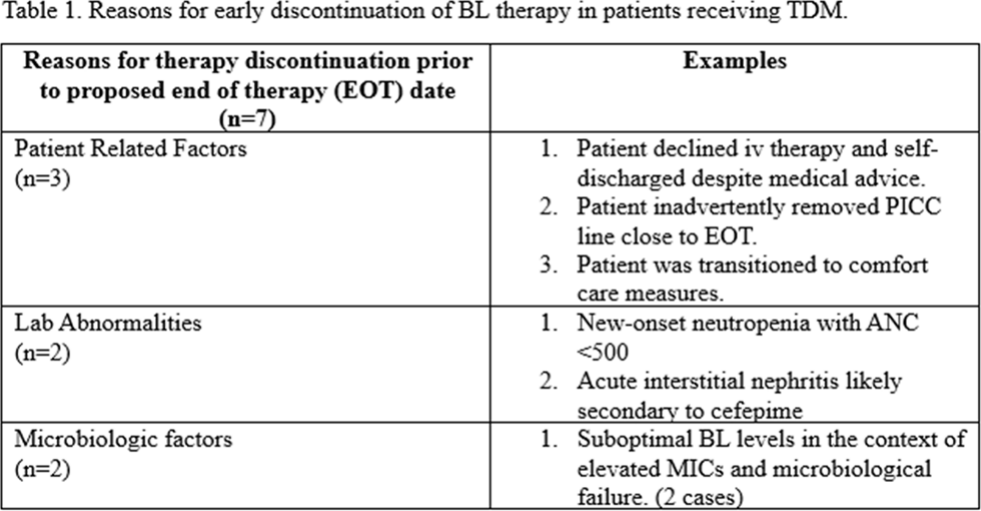

**Results:**

During the study period, 25 OPAT patients underwent BL TDM. TDM was performed in patients receiving CEF (64%), TZP (20%), and MEN (16%). The most common indication for TDM was suspected beta-lactam toxicity (32%) (Figure 1). Post-TDM therapy adjustments were made in 52% (n = 13), including changes in dosing frequency (38.5%) and dosage strength (30.8%). Additional changes included infusion strategy modification or agent substitution (15.4% each). Subtherapeutic levels were the most common reason for adjustment (46.2%), followed by adverse effects, supratherapeutic levels, and changes in renal function (23.1% each).

Skin, soft tissue, and musculoskeletal infections were most common (Figure 2). Positive cultures were present in 80%, with *Pseudomonas* spp most frequently isolated. Therapy was completed in 17 patients; 7 discontinued early, and 1 had treatment ongoing. Reasons for therapy discontinuation are detailed in Table 1.

**Conclusion:**

BL TDM is contributing to a paradigm shift in OPAT practice by improving both efficacy and safety. While inpatient TDM typically targets efficacy, safety concerns more commonly motivate TDM in the OPAT setting. Although this review involved a small cohort, our experience adds to the growing body of evidence supporting BL TDM as a tool to enhance patient outcomes in the OPAT setting.

**Disclosures:**

All Authors: No reported disclosures

